# Analyzing Data Modalities for Cattle Weight Estimation Using Deep Learning Models

**DOI:** 10.3390/jimaging10030072

**Published:** 2024-03-21

**Authors:** Hina Afridi, Mohib Ullah, Øyvind Nordbø, Solvei Cottis Hoff, Siri Furre, Anne Guro Larsgard, Faouzi Alaya Cheikh

**Affiliations:** 1 Department of Computer Science, Norwegian University of Science and Technology, 2815 Gjøvik, Norway; faouzi.cheikh@ntnu.no; 2 Geno SA, Storhamargata 44, 2317 Hamar, Norway; anne.guro.larsgard@geno.no; 3 Norsvin SA, Storhamargata 44, 2317 Hamar, Norway; oyvind.nordbo@norsvin.no; 4 TYR SA, Storhamargata 44, 2317 Hamar, Norway; solvei@tyr.no (S.C.H.); siri.furre@tyr.no (S.F.)

**Keywords:** cattle weight estimation, data modalities, depth information, segmentation, deep learning models

## Abstract

We investigate the impact of different data modalities for cattle weight estimation. For this purpose, we collect and present our own cattle dataset representing the data modalities: RGB, depth, combined RGB and depth, segmentation, and combined segmentation and depth information. We explore a recent vision-transformer-based zero-shot model proposed by Meta AI Research for producing the segmentation data modality and for extracting the cattle-only region from the images. For experimental analysis, we consider three baseline deep learning models. The objective is to assess how the integration of diverse data sources influences the accuracy and robustness of the deep learning models considering four different performance metrics: mean absolute error (MAE), root mean squared error (RMSE), mean absolute percentage error (MAPE), and *R*-squared ($R^{2}$). We explore the synergies and challenges associated with each modality and their combined use in enhancing the precision of cattle weight prediction. Through comprehensive experimentation and evaluation, we aim to provide insights into the effectiveness of different data modalities in improving the performance of established deep learning models, facilitating informed decision-making for precision livestock management systems.

## 1. Introduction

**Figure 1:**
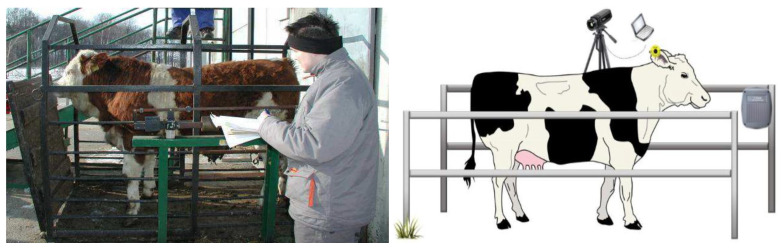
On the (**left side**), the weighing of cattle on a scale is shown, where a person is manually noting the corresponding value on paper. On the (**right side**), the weight of the cattle is automatically estimated via camera sensors.

Estimating the weight of cattle is essential for sustainable beef production and cattle breeding since it helps the farmer track the growth of the cattle, [1]. Precise weight estimation plays a crucial role in well-informed decision-making [2] regarding slaughtering, safeguarding the welfare of cattle [3], and maximizing beef production. Weight estimation has historically been done using manual methods [4] (as shown on the left side of Figure 1) that do not have the accuracy needed for contemporary farming techniques [5]. Manual methods are time-consuming and stressful because the cattle are physically handled and fixated. Thus, they are not good for cattle welfare. The shortcomings of these manual techniques highlight the need for an automated process that embraces the cutting-edge capabilities provided by cutting-edge technologies [6], especially in the areas of artificial intelligence and machine learning [7] (as shown on the right side of Figure 1).

In recent years, we have seen encouraging outcomes from the integration of deep learning techniques in a number of fields, such as computer vision [8] and natural language processing [9]. With the development of deep learning, there is now an opportunity to use complicated deep models to predict weight more accurately and automatically. However, this problem presents a challenge due to the complexity of predicting weights when converting 3-D space to 2-D images. This conversion results in the loss of the cattle shape information. In our work, we investigate 2-D RGB images and depth images, specifically focusing on the effects of only-cattle regions and various data modalities [10] on prediction accuracy. To further elaborate, the only-cattle region represents images where the RGB values representing cattle are preserved but the rest of the values are set to 0. The purpose is to see how the models trained on such images would perform. Our research highlights how important it is to take advantage of different data modalities in order to improve the functionality of current deep learning models for cattle weight estimation. For this purpose, we collect RGB images (RGB) and depth images (DP) of cattle from different farms. We also combine these two data sources to produce a third data modality, namely, the RGBD data modality. We explore the recent vision-transformer-based zero-shot model [11] from Meta AI Research to generate a fourth data modality called fully segmented images (FS). We also combine the FS images with the DP images to generate a fifth data modality namely, fully segmented images with depth information (FSD).

The exploration of different data modalities is motivated by the need to understand their individual and collective impact on the performance of different baseline deep learning models including the Inception V3 (INC) model [12], the MobileNet (MOB) model [13], and the EfficientNet B1 (EFF) model [14]. Our research contributes to the broader field of cattle management, presenting important insights for practitioners seeking to use reliable cattle weight estimation systems [5]. We specifically consider RGB imagery, depth information, combined RGB and depth data, and segmented information as distinct modalities for cattle weight estimation. Each modality presents distinctive insights into the physical characteristics and spatial relationships of the cattle. By evaluating the performance of three baseline deep learning models across these modalities, we focus on understanding the impact of diverse data sources on the accuracy and robustness of cattle weight predictions. The main contributions of this paper are:

- To the best of our knowledge, we are the first to explore the vision-transformer-based zero-shot model for producing the only-cattle region and FS data modality. We analyze five different data modalities for cattle weight estimation;
- We investigate the impact of using the only-cattle region from RGB images and five different data modalities on the performances of three famous baseline DL models;
- We collect and present our own cattle dataset consisting of five different data modalities for cattle weight estimation;
- We present comprehensive experiments to evaluate the impact of these data modalities on the performances of the baseline DL models using four different performance metrics: MAE, RMSE, MAPE, and *R*-squared ($R^{2}$).

The rest of the paper is organized as follows. In Section 2, an overview of related works is described. The exploration and usage of the baseline deep learning models are outlined in Section 3. Experimental analysis on our collected cattle dataset is presented in Section 4. We present the discussion in Section 5 and conclusions in Section 6.

## 2. Related Works

This literature review is divided into two main categories. The first category encompasses studies related to conventional methods for cattle weight estimation, and the second category focuses on research pertaining to deep learning methods.

In the first category, the study by Dang et al. [15] investigated the viability of employing ten body measurements as input features to estimate the body live weight of Hanwoo cows. For this purpose, the machine learning models FT-Transformer, TabNet, Light Gradient Boosting Machine, Multilayer Perceptron, and k-Nearest Neighbour are used. The link between the weights and body size measurements (features) of cows is investigated through the use of machine-learning-based data analysis. Weber et al. [16] used regression methods to automatically extract measures from images of the dorsal area of Nellore cattle in order to determine the cattle’s weight. Euclidean distances from locations produced by the active contour model were chosen for this purpose by the authors together with characteristics gleaned from the dorsal Convex Hull. Na et al. [17] used the Bayesian ridge algorithm on RGB-D images for automatic weight prediction of cattle. They performed segmentation, extraction of features [18], and estimation of the weight of cattle using depth and color information. They exploited three features: size, shape, and gradients. Ruchay et al. [19] used the random forest algorithm for cattle weight estimation. They build a relationship between the dependent variable, i.e., body weight, and independent variables such as withers height, hip height, chest depth, and chest width. Alonso et al. [20] used a function to predict the carcass weight of beef cattle by exploiting support vector machines for regression. The function considered a few zoometric measurements of the animals. For cattle weight estimation, Alonso et al. [21] exploited the geometrical relationships of the trajectories of weights over time. They modeled a family of parallel functions that fit the whole dataset of cattle using support vector machines. Gomes et al. [22] developed formulations to predict body and carcass weight and body fat content of bulls using digital images obtained through a Microsoft Kinect device. The Kinect sensor, installed on the top of a cattle chute, was used to take infrared-light–based depth videos. A single frame from the recorded videos was identified and used to check different body measurements, including thorax width, abdomen width, body length, and dorsal area.

In the second category, Lee et al. [23] segmented the animal and background, and weights were estimated using fully and weakly supervised methods [24]. The fully supervised segmentation method used a Mask R-CNN model [25] that learns the ground truth mask generated by labeling. He et al. [26] investigated a live weight estimation method based on a Lightweight High-Resolution Network considering RGB-D images. Class activation mapping supported the development of efficient network heads embracing visual explanation and applicability in practical natural livestock environments. Guvenog et al. [27] estimated the weight of cattle by using stereo vision and semantic segmentation methods [28]. Images of animals were captured from various angles with a stereo setup. The distances of the animals from the camera plane were measured by stereo distance calculation, and the areas covered by the animals in the images were determined by semantic segmentation methods. The work of Kwon et al. [29] estimated the weight of animals in real-time using mesh reconstruction and deep learning. The authors’ approach had two phases. In the first phase, they produced training data by mesh reconstruction from point clouds of animals and modeled a deep neural network to calculate the weight by using the training data. Hou et al. [30] introduced a non-contact body weight estimation with a 3D deep learning model. The three-dimensional (3D) point cloud data of the whole contour surface of a beef cattle are proximally obtained by light detection and a ranging (LiDAR) sensor. However, it is worth noting that LiDAR is an expensive sensor that also needs maintenance over time due to its mechanical movements. Ruchay et al. [31] stated that a promising way to estimate live weight is by considering morphometric measurements of livestock and then applying regression equations affiliated with such measurements. They introduced a model for estimating live weight based on augmenting three-dimensional clouds through flat projections and image regression with deep learning. Meckbach et al. [32] estimated weight based on convolutional neuronal networks. They used only depth images. They presented their work as preliminary research to confirm the ability of using convolutional neural networks for weight estimation. Gjergji et al. [33] studied deep learning models including RNN networks [34], recurrent attention models [35], and recurrent attention models with convolutional neural networks to assess their performances in predicting cattle weight. They found that the convolutional neural networks obtained good results.

Our research involves the analysis of various data modalities for cattle weight estimation through the utilization of baseline deep learning models. Therefore, our work aligns with the second category delineated in the above review.

## 3. Proposed Methodology

We explore the model proposed by Kirrilov et al. [11] to extract the only-cattle region from RGB images and to produce the fourth data modality representing fully segmented images (FS). This model is a novel vision-transformer-based zero-shot model [11] developed by the researchers at Meta AI research. The model is based on the use of a data collection loop to build the largest segmentation dataset to date, with over 1 billion masks on 11 million licensed and privacy-respecting images. The model can transfer zero-shot to new image distributions and tasks. The model is capable of segmenting any object on a certain image. The model produces high quality object masks, which can be explored to produce masks for all objects in an image. It has a strong zero-shot performance on a variety of segmentation tasks.

**Figure 2:**
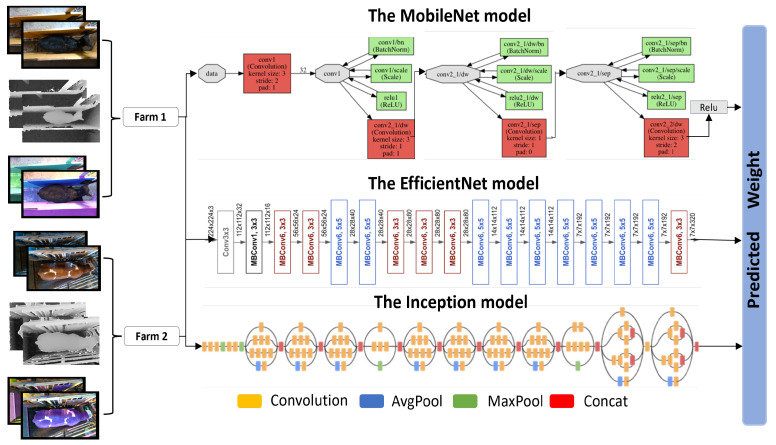
The MobileNet model (MOB), the EfficientNet B1 model (EFF), and the Inception model (INC). The data in different modalities are fed to each model individually, which predict cattle weight after the training process.

For cattle weight estimation using different data modalities, we explore the Inception V3 (INC) model [12], the MobileNet (MOB) model [13], and the EfficientNet B1 (EFF) model [14], as depicted in Figure 2. We utilized the pre-trained architectures of all three models, whereby we froze the initial 15 layers and retrained the rest of the layers with our own collected data. All three models use mean absolute error (MAE) as a loss function for training. It is a simple yet robust measure for evaluating the accuracy of models for regression tasks, that is, in our case, cattle weight estimation. It is formulated as,

(1) $$\begin{array}{c} MAE=\frac{1}{n}\sum_{i=1}^{n}\left|y_{i}-{\hat{y}}_{i}\right| \end{array}$$

where *n* is the number of data points, $y_{i}$ represents the actual target value for data point *i*, and ${\hat{y}}_{i}$ represents the predicted value for data point *i*.

The Inception V3 (INC) model is a deep convolutional neural network. The INC model considers factorized 7 × 7 convolutions, which reduces the computational complexity of the model by breaking down larger convolutions into smaller ones. This decline in computational overhead allows the INC model to cope with a variety of data efficiently. The INC model utilizes label smoothing, a technique to regularize the model by considering the effect of label dropout during the training process. This hinders the model from predicting a value too confidently in term of overfitting. This approach restricts the model from making overly confident predictions, enhancing its capability to generalize to distinct cattle weight patterns. The INC model considers batch normalization extensively. Batch normalization provides support in improving the speed, performance, and stability of the model. The model uses the RMSprop optimizer, which is known for its robustness in handling non-stationary settings, and takes the form,

(2) $$\begin{array}{cc} E{\left[g^{2}\right]}_{t} & =0.9E{\left[g^{2}\right]}_{t-1}+0.1g_{t}^{2}\\ W_{t+1} & =W_{t}-\frac{\eta }{\sqrt{E{\left[g^{2}\right]}_{t}+\epsilon }}g_{t} \end{array}$$

where $E{\left[g^{2}\right]}_{t}$ is the running average of the squared gradient, $g_{t}$ is the gradient, and $W_{t}$ is the weight at time step *t*. η is the learning rate and ϵ is a small constant added to improve numerical stability. This, therefore, makes the INC model suitable for cattle weight estimation, a domain in which the data patterns may vary. The model accommodates diverse data modalities for accurate cattle weight estimation. Through normalization and resizing, RGB, depth, and combined data are seamlessly integrated into the model. The INC model accommodates multiple input channels, allowing each modality to contribute its unique information. The INC model efficiently combines features extracted from distinct modalities and ensures alignment with the intricacies of the cattle weight estimation task. The output layer is configured to provide regression outputs, culminating in an effective framework for cattle weight prediction across varied data modalities.

The MobileNet (MOB) model is also a convolutional neural network. The model uses depthwise separable convolutions, which include depthwise and pointwise convolutions, to significantly reduce the number of parameters, resulting in a lightweight deep neural network. The depthwise convolution and pointwise convolutions are formulated as,

(3) $$\begin{array}{c} Y_{i,j,k}=\sum_{m,n}X_{i+m,j+n,k}\times K_{m,n,k}\\ Z_{i,j,l}=\sum_{k}Y_{i,j,k}\times L_{k,l} \end{array}$$

where *Y* is the output feature map, *X* is the input feature map, *K* is the depthwise kernel, *i* and *j* are spatial indices, the variables *m* and *n* represent the spatial dimensions used for convolution operations, *k* is the channel index, *L* is the pointwise kernel, and *l* is the output channel index. The MOB model is a good architecture for cattle weight estimation due to the proper encoding and learning of distinct patterns from diverse data modalities. Its depthwise separable convolutions significantly reduce computational complexity, making it well-suited for resource-constrained environments or real-time applications. In the context of cattle weight estimation, where processing diverse data modalities like RGB, depth, and combined data is essential, the adaptability of the MOB model for multi-channel inputs ensures seamless integration. The model accommodates multi-channel inputs, enabling each modality to contribute its unique information. Depthwise-separable convolutions and feature concatenation ensure effective integration of information from different modalities. The model learns to discern the nuances associated with each modality. The output layer performs regression for the cattle weight estimation.

The EfficientNet B1 (EFF) model is a convolutional neural network that uses a compound scaling method. The model uniformly scales all dimensions of depth, width, and resolution, resulting in improved representation power to achieve better performance. In the EFF model, the width multiplier controls the number of channels in each layer and the depth multiplier regulates the number of layers in the network. The number of layers in the network is scaled by the depth multiplier, denoted as *d*. If *L* is the original number of layers, then the new number of layers $L^{\prime }$ is given by:

(4) $$\begin{array}{c} L^{\prime }=L\times d \end{array}$$

The number of channels in each layer is scaled by the width multiplier, denoted as *w*. If *C* is the original number of channels, then the new number of channels $C^{\prime }$ is given by:

(5) $$\begin{array}{c} C^{\prime }=C\times w \end{array}$$

The resolution of the input image is scaled by the resolution multiplier, denoted as *r*. If *S* is the original resolution, then the new resolution $S^{\prime }$ is given by:

(6) $$\begin{array}{c} S^{\prime }=S\times r \end{array}$$

The scalability of the EFF model enables it to effectively handle diverse data modalities, including RGB, depth, and segmented information relevant to cattle weight estimation. The strength of the model’s ability to adapt its architecture based on the characteristics of the input data makes it particularly efficient in capturing intricate patterns from varied sources. Therefore, the EFF model can handle complex patterns in images from RGB cameras, depth information, and segmented data. The model aligns well with the multi-modal nature of cattle weight estimation. The model learns and extracts meaningful features from different sources, contributing to accurate cattle weight prediction. Therefore, in the context of cattle weight estimation, the feature-rich architecture of the EFF model ensures seamless integration and processing of cattle data, contributing to its versatility and effectiveness in addressing the complexities of cattle weight prediction.

## 4. Experimental Results

**Figure 3:**
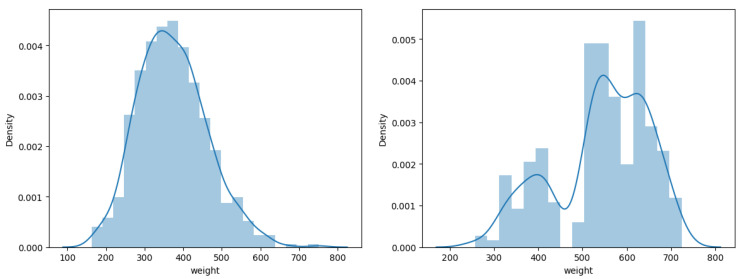
Distribution of data: the distribution of data in Farm 1 (**left side**) and Farm 2 (**right side**).

**Figure 4:**
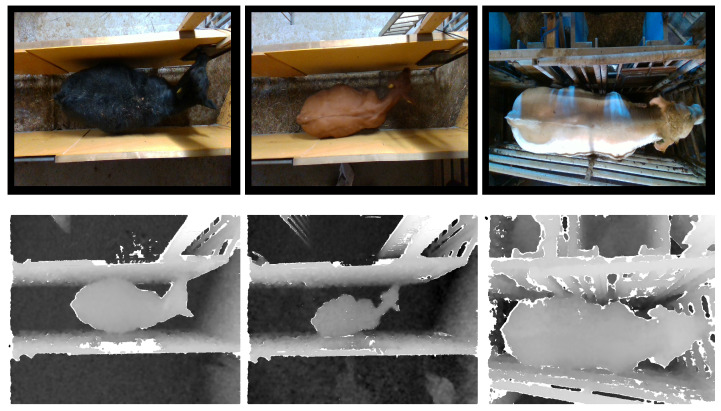
Dataset: Some sample images from both Farm 1 and Farm 2. The (**first row**) shows RGB images, and the (**second row**) shows the corresponding depth images.

**Figure 5:**
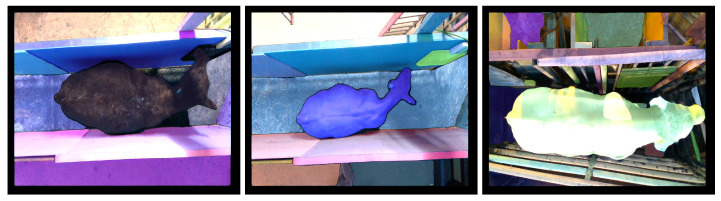
Results of segmentation: the results of segmentation for sample images from Farm 1 and Farm 2.

We captured the cattle images used in this study using an Intel RealSense D415 camera. The camera has a standard field of view well suited for capturing cattle images. We installed customized software to capture the images of the cattle. The camera exploits rolling shutter sensors, which present high depth quality per degree. The camera also includes an IR pattern projector to illuminate environments with poor lighting. It is also worth noticing that the RGB sensor on the D415 has a very good low-light sensitivity to reduce blurring during fast motion indoors under most normal lighting conditions. Using the camera, the images were captured from two different beef cattle farms. For the sake of simplicity, we will refer to them as Farm 1 and Farm 2. The dataset from Farm 1 comprises a collection of 613 RGB images (RGB) and the corresponding 613 depth images (DP). This dataset predominantly features black cattle. The controlled environment in this farm maintains consistency, limiting factors such as blurriness caused by animal movement. Meanwhile, Farm 2 contributes 676 RGB images (RGB) and the corresponding 676 depth images (DP). The dataset from Farm 2 showcases a diverse mix of cattle in both white and brown hues. Unlike Farm 1, the weighing station in Farm 2 introduces variability due to the irregular conditions of door open/close scenarios and fluctuating lighting conditions. We present the distribution of images in Farm 1 and Farm 2 in Figure 3. In combination, these datasets provide a comprehensive set of images capturing different aspects of cattle appearance and environmental conditions for a holistic analysis. We provide sample images from both Farm 1 and Farm 2 in Figure 4. We further processed the data from Farm 1 and Farm 2 to merge the RGB images (RGB) with the corresponding depth images (DP). With this merging process, we obtain the third modality of data, which we call (RGBD) data. To generate the fourth data modality, we explore the recent vision-transformer-based zero-shot model [11] from Meta AI Research. We consider only the pre-trained model. The fourth data modality represents the fully segmented images (FS), for which we provided only RGB images as input to the model. We present sample FS images for both Farm 1 and Farm 2 in Figure 5. We also combined the FS images with the depth images (DP) to generate the fifth data modality. We call these fully segmented images with depth information (FSD). We also explore the vision-transformer-based zero-shot model [11] to extract the only-cattle region from the RGB images of Farm 1. The purpose of this is to analyze how the only-cattle region contributes to the performances of the models for cattle weight estimation.

For experimental analysis, we consider the performance metrics mean absolute error (MAE), root mean squared error (RMSE), mean absolute percentage error (MAPE), and *R*-squared ($R^{2}$). MAE represents the average absolute difference between the predicted and actual weights. It provides a straightforward measure of prediction accuracy, making it easy to interpret. RMSE considers the square of the differences between predicted and actual weights, providing more weight to larger errors. It is useful for penalizing significant deviations, giving a sense of the overall model performance. MAPE expresses errors as a percentage of the actual values, providing a relative measure of accuracy. It is particularly valuable when assessing the model’s performance in the context of different cattle weights. ($R^{2}$) measures the proportion of the variance in the dependent variable (cattle weights) that is predictable from the independent variables (features used in the model). A higher ($R^{2}$) suggests that the model explains a larger proportion of the variability in cattle weights. It is a key metric for evaluating the goodness of fit and overall effectiveness of the predictive model. We used 80% data for training and 20% data for validation.

**Figure 6:**
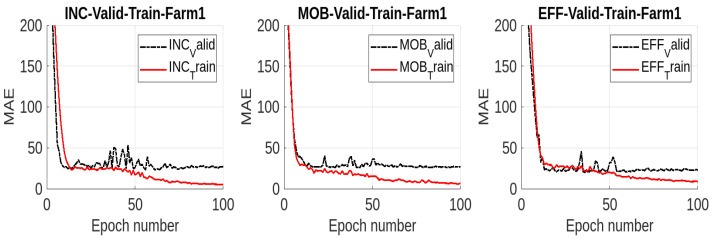
Results for the only-cattle region from RGB images from Farm1 using MAE: training and validation results using mean absolute error (MAE) for all three models.

We present the results for considering the only-cattle region from RGB images from Farm 1 in Figure 6. The results are shown for all the three models INC, MOB, and EFF. As can be seen, all the models become overfitted by considering only the cattle region. We also report the performances of three deep learning models using the performance metrics mean absolute error (MAE), root mean squared error (RMSE), mean absolute percentage error (MAPE), and *R*-squared ($R^{2}$) in Table 1. The values for these metrics are unsatisfactory for all the models, representing poor performances. In fact, the region-specific analysis related to cattle does not capture the comprehensive features necessary for accurate weight prediction. Additionally, the context of the surrounding environment helps models better encode the features related to weight estimation. Therefore, the analysis results in a limited and biased representation of the data by overlooking these contextual cues. Based on this analysis, we will not use the only-cattle region for further analysis. The focus of the analysis will instead be on the five data modalities we mentioned earlier.

**Figure 7:**
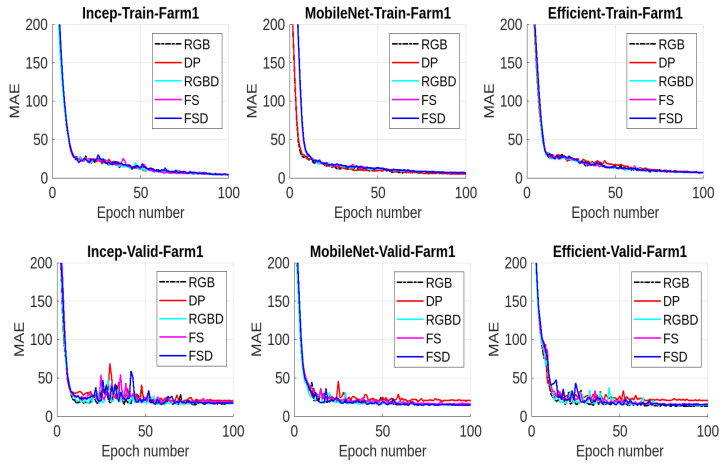

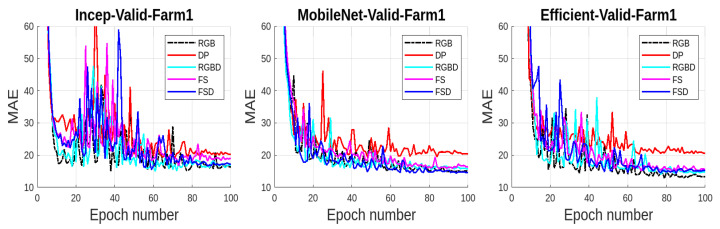
Results from Farm1 using MAE: In the (**top row**), we present the training results using mean absolute error (MAE) for all three models considering five data modalities. In the (**middle row**), we present the validation results. In the (**bottom row**), we present the enlarged validation results for better visualization.

**Figure 8:**
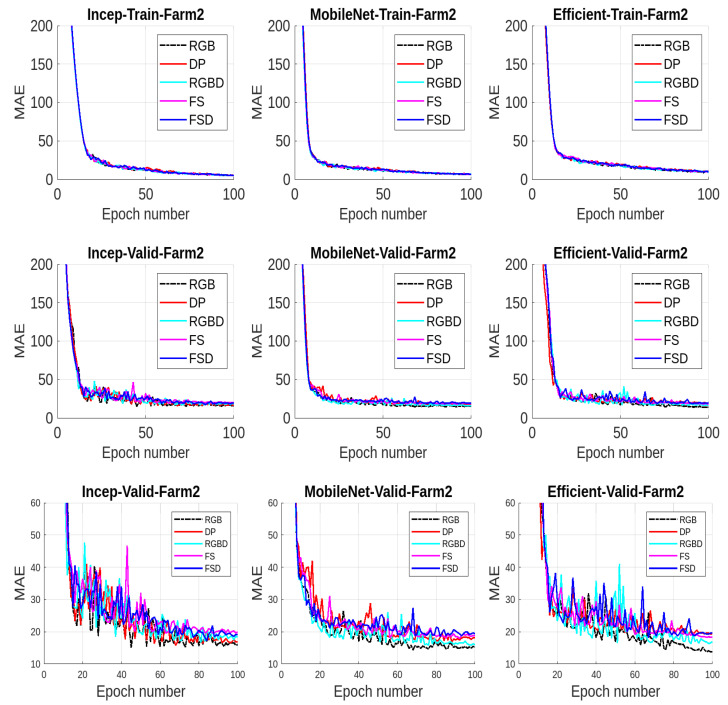
Results from Farm 2 using MAE: (**Top row**): training results using mean absolute error (MAE) for all three models considering all five data modalities. (**Middle row**): validation results. (**Bottom row**): enlarged validation results for better visualization.

**Figure 9:**
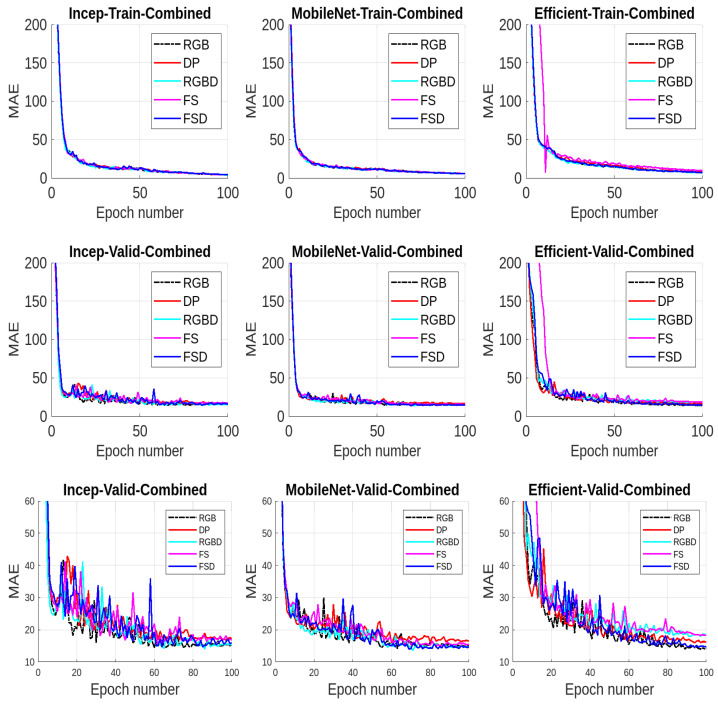
Results from combined dataset (Farm 1 and Farm 2) using MAE: In the (**top row**), we present the training results using mean absolute error (MAE) for all three models considering five data modalities. In the (**middle row**), we present the validation results. In the (**bottom row**), we present the enlarged validation results for better visualization.

We present the results for all the five data modalities for Farm 1 in Figure 7, considering the three models INC, MOB, and EFF. In this analysis, three deep learning models, INC, MOB, and EFF, are evaluated across five distinct data modalities to assess their performance in cattle weight estimation. Through this analysis across the RGB, DP, RGBD, FS, and FSD modalities, we aim to uncover how each model adapts to and leverages diverse data characteristics for accurate cattle weight estimation. The INC and MOB models present good performances considering the MAE performance metric. However, the EFF model, characterized by uniform scaling of depth, width, and resolution, offers a scalable architecture for addressing different complexities in these data modalities and has even better performance than INC and MOB. The model shows stable output considering the RGB, RGBD, and FSD modalities. In Figure 8, we present the results for all five data modalities for Farm 2, considering the three models INC, MOB, and EFF. Furthermore, in this case, we evaluate these models across these data modalities to analyze their performances using the MAE metric. As can be seen, the situation remains the same as with Farm 1. Again, the EFF model presents better results considering these data modalities. Furthermore, in Figure 9, we present the results for all five data modalities by combining data from both Farm 1 and Farm 2. In this analysis encompassing the RGB, DP, RGBD, FS, and FSD modalities, our primary objective is to elucidate how each model adapts to and harnesses diverse data characteristics for precise cattle weight estimation. Notably, all three models demonstrate commendable performance levels. The robustness of their performance across varied data modalities positions these models as promising candidates for accurate and reliable cattle weight predictions. As can be seen, the variations in the MAE score are not very pronounced in the case of the EFF model.

### Ablation Study

**Figure 10:**
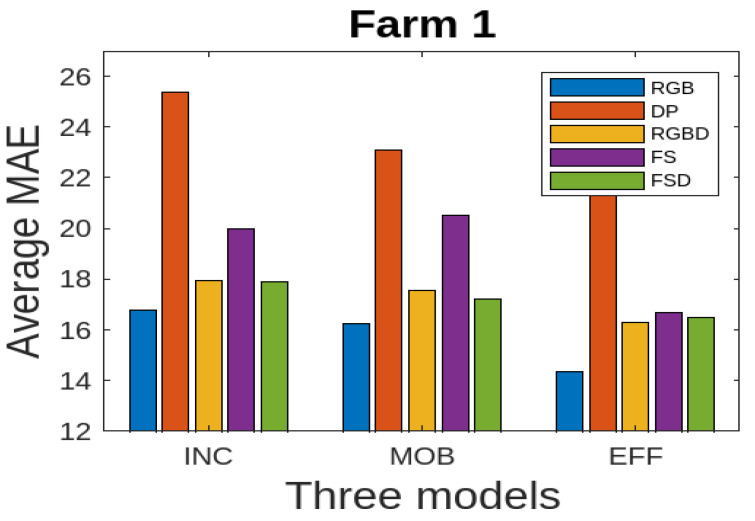
Farm 1: average MAE for three deep learning models using five different data modalities.

For further experimental analysis, we report the results for all data modalities for Farm 1, in Table 2, considering the performance metrics MAE, RMSE, MAPE, and ($R^{2}$). The MAE metric results reveal that the RGB, RGBD, and FSD modalities demonstrate commendable predictive accuracy, with consistently low absolute differences between predicted and actual weights. The results for the RMSE metric further affirm the robustness of these data modalities considering all three models. The MAPE metric results highlight the proportional accuracy, showcasing the ability of the models to maintain precision across varying weight scales. Additionally, the ($R^{2}$) metric results elucidate the extent to which each modality explains the variance in cattle weights, highlighting the modalities that effectively captured the dataset’s variability. We also report the results of three models using MAE in Figure 10. This also shows that RGB, RGBD, and FSD represent good accuracies. This comprehensive approach to performance evaluation facilitates a nuanced understanding of the strengths and weaknesses inherent in each data modality, contributing to informed decision-making in the realm of cattle weight estimation.

**Figure 11:**
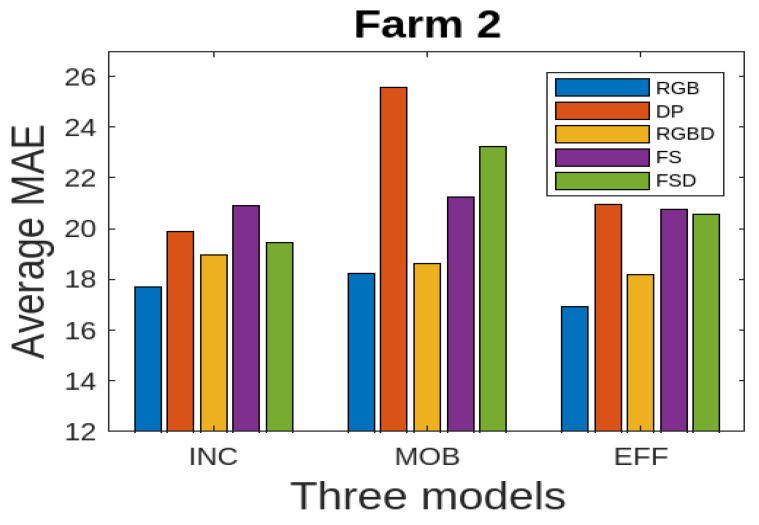
Farm 2: average MAE for three deep learning models using five different data modalities.

In Table 3, we report the results for all data modalities for Farm 2. The MAE metric results indicate that the RGB, RGBD, and FSD modalities exhibit significantly good predictive accuracy, as evidenced by consistently small absolute differences between predicted and actual weights. The robustness of these data modalities is further confirmed by the RMSE metric results across all three models. The results for the MAPE metric underscore the proportional accuracy, demonstrating the capacity of the models to maintain precision across different weight scales. The ($R^{2}$) metric results shed light on the degree to which each modality accounts for the variance in cattle weights, emphasizing the modalities that effectively encapsulated the variability of the dataset. We also report the results of three models using MAE in Figure 11. It also shows that RGB, RGBD, and FSD represent good accuracies.

**Figure 12:**
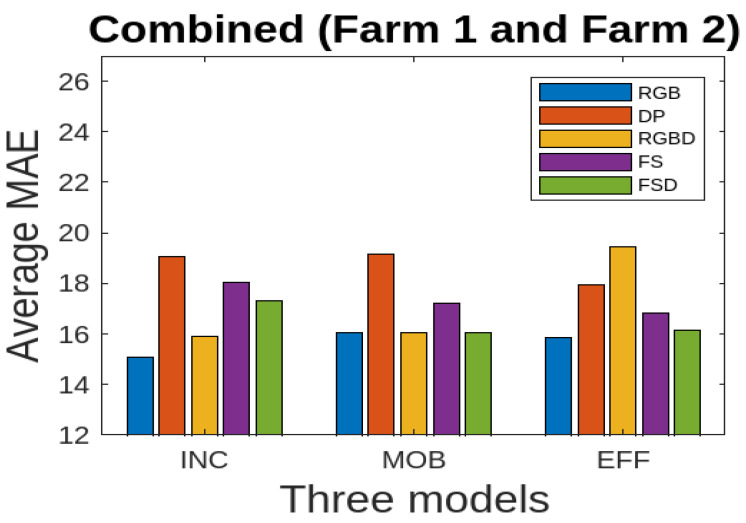
Combined data (Farm 1 and Farm 2): average MAE for three deep learning models using five different data modalities.

Moreover, in Table 4, we present the results for the combined data from Farm 1 and Farm 2. Here, the compelling analysis of cattle weight estimation across diverse data modalities brings forth noteworthy findings. The MAE metric results unequivocally highlight the superior predictive accuracy of the RGB, RGBD, and FSD modalities, showcasing their consistent ability to yield minimal absolute differences between predicted and actual weights. This resilience is further underscored by the results for the RMSE metric, reaffirming the robust nature of these modalities across all three models. Delving into proportional accuracy, the MAPE metric results accentuate the precision upheld by the models, demonstrating their adeptness at maintaining accuracy irrespective of varying weight scales. Moreover, the ($R^{2}$) metric results offer a profound insight into the capability of the modalities to elucidate the variance in cattle weights, pinpointing those that effectively capture the intricate dataset variability. We also report the results of three models using MAE in Figure 12. This also shows that RGB, RGBD, and FSD represent good accuracies. This holistic approach to performance evaluation has enabled a detailed understanding of the inherent characteristics of each data modality.

**Figure 13:**
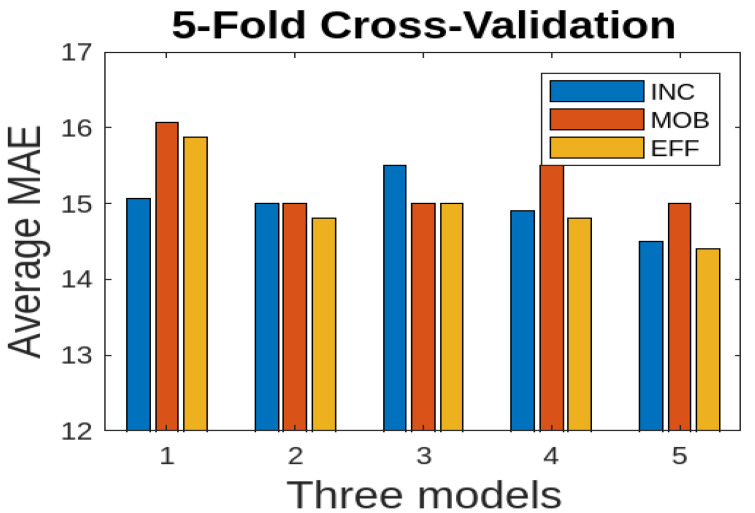
Cross validation for combined data (Farm 1 and Farm 2): average MAE for three deep learning models using the RGB modality.

**Figure 14:**
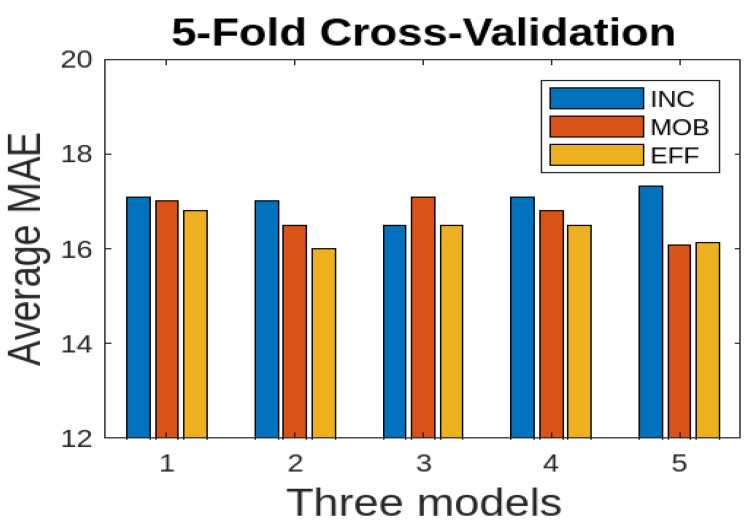
Cross validation for combined data (Farm 1 and Farm 2): average MAE for three deep learning models using the FSD modality.

We also consider 5-fold cross-validation where the dataset is partitioned into five folds, ensuring an equal distribution of samples across folds. We used the average MAE to report the results for three deep learning models. We consider only RGB and FSD data modalities for the combined data (Farm 1 and Farm 2) since they show good results. The results are presented in Figure 13 for only the RGB data modality. The EFF model performs better comparatively; however, there are no significant variations across different folds. For 5-fold cross validation, we also consider the FSD data modality for the combined data (Farm 1 and Farm 2). The results are presented in Figure 14. Here, the EFF model performs better comparatively; however, there are no significant variations across different folds.

**Figure 15:**
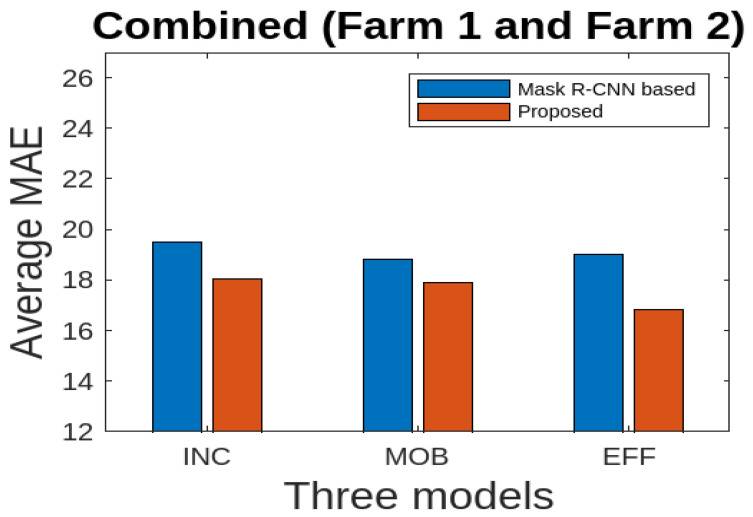
Comparison considering the combined data (Farm 1 and Farm 2): comparison with the reference method using the average MAE for three deep learning models using the FSD modality.

We also perform a comparison with a recent method [23] based on Mask R-CNN. We created fully segmented images (FS) with the compared method [23]. We used only combined data (Farm 1 and Farm 2) for the purpose of comparison. We then used the three deep learning models for cattle weight estimation using the FS modality of the compared method [23], and then our explored method [11]. The results are presented in Figure 15. As can be seen, the performance of our explored method [11] is better in all the three cases.

## 5. Discussion

Initially, we considered the only-cattle region from RGB images from Farm 1. All three baseline models became overfitted with poor performances in terms of other performance metrics. Our research revealed that the region-specific analysis related to cattle does not capture the comprehensive features necessary for proper weight estimation. The context of the surrounding environment also supports the models, allowing them to encode the layout of the physical scene for cattle weight estimation. Subsequently, we considered multiple data modalities, such as RGB, depth, RGBD, segmentation data, and the combination of segmentation and depth data in the complex field of cattle weight estimation. Through experimental analysis, we investigated data patterns to show the complex dynamics of data modalities and how they affect the performances of deep learning models when it comes to estimating the weight of cattle. The models considering only the RGB data present good performance, highlighting the value of color-based data in collecting crucial aspects for weight prediction. The models also present good performances by considering the RGBD data modality, where depth-sensing provides additional spatial information for better weight estimation.

However, the models relying only on depth information for cattle weight estimation fall short of delivering optimal performance due to the inherent limitations of depth data alone. Depth information provides a representation of the spatial distances between objects in a scene, offering a valuable three-dimensional perspective. However, in our context, where we have images only from the top view, this modality encounters challenges that impede its standalone efficacy. The intrinsic variety in cattle appearance and pose is one of the main limitations. Cattle can have a wide range of sizes, shapes, and positions in the image, which can result in intricate and erratic depth patterns. The ability of the depth modality to differentiate between various cow features is further limited by the absence of color information. Additionally, noise and inconsistencies in depth data can cause uncertainties, particularly in situations when lighting is poor, or cattle are in close proximity. These considerations lead to the limited discriminative capability of depth information alone.

On the other hand, the models could perform better if the depth information is combined with other modalities like RGB or segmentation. The performance of each model is improved when depth is combined with colour information or spatial context from segmentation, producing a distinct representation for weight prediction. The borders and spatial distribution of the cattle in the images are taken into consideration by these modalities, adding some level of context. Some of these modalities could lead to more accurate weight estimation if a very large amount of data is available. Considering the segmentation data without depth, on the other hand, does not yield the same level of performance. This suggests that the depth modality is crucial for improving weight estimation since it provides depth signals that aid in determining the structure of the cattle. However, this small indication, in our case, creates opportunities for future advancements in the use of diverse data modalities for precision agriculture applications.

## 6. Conclusions

In this work, we investigated different data modalities for cattle weight estimation using three baseline deep learning models. We also collected and presented our own cattle dataset with four different modalities. We generated an additional data modality through the vision-transformer-based zero-shot model. We performed experimental analysis using the performance metrics MAE, RMSE, MAPE, and *R*-Squared, to analyze how the combination of diverse data sources impacts the accuracy of the models. Our work showed that both RGB and RGBD models perform well. However, a more robust cattle weight estimation can be obtained by merging segmentation and depth depending on the amount of data and the variations in the data.

In our future work, we would like to collect more data and analyze how depth information, in combination with other data modalities, would impact the performance of the models. Moreover, a comprehensive examination of the potential integration of depth information with the binary mask produced by segmentation to enhance weight estimation remains unfinished. This is especially crucial because using the cattle area modality alone has resulted in overfitting problems.

## Figures and Tables

**Table 1:** Only-cattle region from Farm 1: Performance of three deep learning models considering only the cattle region in images from Farm 1 using four different performance metrics, mean absolute error (MAE), root mean squared error (RMSE), mean absolute percentage error (MAPE), and *R*-squared ($R^{2}$). The MAE and RMSE errors can be interpreted as average errors in kilograms between the predicted and actual cattle weights, while the MAPE and *R*-Squared metrics represent the accuracy and goodness-of-fit of the models and do not have a direct interpretation in kilograms.

Models	MAE (Kg)	RMSE (Kg)	MAPE (%)	*R*-Squared ($R^{2}$)
INC	35.54	43.42	9.35	0.73
MOB	32.78	40.86	8.87	0.76
EFF	26.53	34.63	7.12	0.82

**Table 2:** Farm 1: Performance of three deep learning models considering all the data modalities using four different performance metrics, mean absolute error (MAE), root mean squared error (RMSE), mean absolute percentage error (MAPE), and *R*-squared ($R^{2}$). The MAE and RMSE errors can be interpreted as average errors in kilograms between the predicted and actual cattle weights, while the MAPE and *R*-Squared metrics represent the accuracy and goodness-of-fit of the models and do not have a direct interpretation in kilograms.

Model	Data Modalities	MAE (Kg)	RMSE (Kg)	MAPE (%)	*R*-Squared ($R^{2}$)
INC	RGB	16.80	24.18	4.24	0.94
	DP	25.35	32.96	6.18	0.88
	RGBD	17.93	26.43	4.58	0.93
	FS	20.00	29.23	5.12	0.91
	FSD	17.91	24.39	4.58	0.90
MOB	RGB	16.24	22.74	4.38	0.95
	DP	23.09	31.86	6.03	0.89
	RGBD	17.56	23.78	4.56	0.94
	FS	20.54	29.47	5.08	0.91
	FSD	17.20	24.12	4.31	0.90
EFF	RGB	14.35	19.53	3.99	0.96
	DP	24.32	30.87	6.60	0.90
	RGBD	16.32	20.94	4.29	0.95
	FS	16.67	24.09	4.43	0.94
	FSD	16.48	20.59	4.58	0.93

**Table 3:** Farm 2: Performance of three deep learning models considering all the data modalities using four different performance metrics, including mean absolute error (MAE), root mean squared error (RMSE), mean absolute percentage error (MAPE), and *R*-squared ($R^{2}$). The MAE and RMSE errors can be interpreted as average errors in kilograms between the predicted and actual cattle weights, while the MAPE and *R*-Squared metrics represent the accuracy and goodness-of-fit of the models and do not have a direct interpretation in kilograms.

Model	Data Modalities	MAE (Kg)	RMSE (Kg)	MAPE (%)	*R*-Squared ($R^{2}$)
INC	RGB	17.17	22.66	3.31	0.94
	DP	19.88	28.31	3.88	0.92
	RGBD	18.97	34.51	3.59	0.87
	FS	20.91	28.29	3.92	0.92
	FSD	19.44	25.98	3.79	0.93
MOB	RGB	18.23	24.19	3.49	0.94
	DP	25.57	33.35	4.89	0.89
	RGBD	18.64	32.73	3.54	0.88
	FS	21.26	28.13	3.95	0.92
	FSD	23.26	29.93	4.44	0.90
EFF	RGB	16.94	22.79	3.29	0.94
	DP	20.97	26.48	4.07	0.93
	RGBD	18.17	31.92	3.59	0.89
	FS	20.77	27.73	4.05	0.92
	FSD	20.57	27.14	4.09	0.92

**Table 4:** Combined data (Farm 1 and Farm 2): Performance of three deep learning models considering all the data modalities using four different performance metrics, mean absolute error (MAE), root mean squared error (RMSE), mean absolute percentage error (MAPE), and *R*-squared ($R^{2}$). The MAE and RMSE errors can be interpreted as average errors in kilograms between the predicted and actual cattle weights, while the MAPE and *R*-Squared metrics represent the accuracy and goodness-of-fit of the models and do not have a direct interpretation in kilograms.

Model	Data Modalities	MAE (Kg)	RMSE (Kg)	MAPE (%)	*R*-Squared ($R^{2}$)
INC	RGB	15.06	20.01	3.57	0.97
	DP	19.07	24.73	4.52	0.96
	RGBD	15.89	21.52	3.50	0.97
	FS	18.05	22.47	4.34	0.97
	FSD	17.33	22.96	4.03	0.96
MOB	RGB	16.07	20.36	3.82	0.97
	DP	19.15	25.79	4.67	0.96
	RGBD	16.04	20.64	3.52	0.97
	FS	17.24	22.52	3.91	0.97
	FSD	16.07	21.92	3.69	0.97
EFF	RGB	15.88	21.21	3.79	0.97
	DP	17.95	24.0	4.26	0.96
	RGBD	19.43	38.14	4.24	0.92
	FS	16.85	22.07	4.25	0.97
	FSD	16.13	22.37	3.90	0.97

## Data Availability

The data presented in this study are available on request from the co-author Mohib Ullah (mohib.ullah@ntnu.no). The data are not publicly available due to its proprietary nature and ethical concerns.
